# Spatial Differences in East Scotia Ridge Hydrothermal Vent Food Webs: Influences of Chemistry, Microbiology and Predation on Trophodynamics

**DOI:** 10.1371/journal.pone.0065553

**Published:** 2013-06-07

**Authors:** William D. K. Reid, Christopher J. Sweeting, Ben D. Wigham, Katrin Zwirglmaier, Jeffrey A. Hawkes, Rona A. R. McGill, Katrin Linse, Nicholas V. C. Polunin

**Affiliations:** 1 School of Marine Science and Technology, Newcastle University, Newcastle upon Tyne, United Kingdom; 2 Dove Marine Laboratory, School of Marine Science and Technology, Newcastle University, Cullercoats, United Kingdom; 3 British Antarctic Survey, Natural Environment Research Council, High Cross, Madingley Road, Cambridge, United Kingdom; 4 Ocean and Earth Science, University of Southampton, National Oceanography Centre Southampton, Southampton, United Kingdom; 5 Natural Environment Research Council Life Sciences Mass Spectrometry Facility, Scottish Universities Environmental Research Centre, East Kilbride, United Kingdom; National Institute of Water & Atmospheric Research, New Zealand

## Abstract

The hydrothermal vents on the East Scotia Ridge are the first to be explored in the Antarctic and are dominated by large peltospiroid gastropods, stalked barnacles (*Vulcanolepas* sp.) and anomuran crabs (*Kiwa* sp.) but their food webs are unknown. Vent fluid and macroconsumer samples were collected at three vent sites (E2, E9N and E9S) at distances of tens of metres to hundreds of kilometres apart with contrasting vent fluid chemistries to describe trophic interactions and identify potential carbon fixation pathways using stable isotopes. δ^13^C of dissolved inorganic carbon from vent fluids ranged from −4.6‰ to 0.8‰ at E2 and from −4.4‰ to 1.5‰ at E9. The lowest macroconsumer δ^13^C was observed in peltospiroid gastropods (−30.0‰ to −31.1‰) and indicated carbon fixation via the Calvin-Benson-Bassham (CBB) cycle by endosymbiotic gamma-Proteobacteria. Highest δ^13^C occurred in *Kiwa* sp. (−19.0‰ to −10.5‰), similar to that of the epibionts sampled from their ventral setae. *Kiwa* sp. δ^13^C differed among sites, which were attributed to spatial differences in the epibiont community and the relative contribution of carbon fixed via the reductive tricarboxylic acid (rTCA) and CBB cycles assimilated by *Kiwa* sp. Site differences in carbon fixation pathways were traced into higher trophic levels e.g. a stichasterid asteroid that predates on *Kiwa* sp. Sponges and anemones at the periphery of E2 assimilated a proportion of epipelagic photosynthetic primary production but this was not observed at E9N. Differences in the δ^13^C and δ^34^S values of vent macroconsumers between E2 and E9 sites suggest the relative contributions of photosynthetic and chemoautotrophic carbon fixation (rTCA v CBB) entering the hydrothermal vent food webs vary between the sites.

## Introduction

Deep-sea hydrothermal vents are chemically reducing habitats occurring on mid-ocean and back-arc spreading centres, seamounts, volcanic hotspots and off-axis ridge settings [1], [2], [3]. They are distinct from the surrounding deep sea with respect to environmental conditions, the energy sources sustaining life and their biological communities [4], [5]. High densities of organisms are found to thrive at the interface where hot, mineral-rich fluids discharge from the seafloor and mix with colder, oxygenated seawater. The hot fluids emitted from the seafloor may differ in pH and are enriched in reduced gases (e.g. H_2_S, CH_4_, H_2_) and metals (e.g. Fe^2+^, Cu, Mn) relative to seawater [6]. Microorganisms oxidise the reduced species in vent fluids and utilise the energy released to fix CO_2_ or other single carbon compounds (e.g. CO, CH_4_) into cellular material [7]. This results in microbial chemosynthesis replacing photosynthetic primary production at the base of the food chain [7].

Sulfide oxidation appears to be the principal energy acquisition pathway, which microorganisms use to drive carbon fixation [3], [7], [8]. The most important carbon fixation pathways at the base of the metazoan hydrothermal vent food webs are the Calvin-Benson-Bassham (CBB) and reductive tricarboxylic acid (rTCA) cycles [9], [10], [11]. Methane oxidation (methanotrophy) is a further carbon fixation process at hydrothermal vents with CH_4_ of thermogenic, biogenic or magmatic origin available depending on the host substrate [3], [12]. Epipelagic photosynthetic primary production may also provide some nutrition to vent macroconsumers, although the relative contribution to vent fauna is thought to be negligible [12], [13]. Macroconsumers utilise the vent organic carbon through endo- and episymbiotic relationships, consumption of free-living microorganisms either from various surfaces or the water column and indirectly through predation and scavenging [14], [15], [16].

The relative contributions of different carbon sources and complexity of hydrothermal vent food webs vary globally depending on the species present, the geological host substrate and the vent fluid chemistry [17], [18], [19]. The first Antarctic hydrothermal vent communities were discovered recently on the East Scotia Ridge (ESR), a back-arc spreading centre in the Atlantic sector of the Southern Ocean [20], [21]. The two basalt-hosted vent fields occur on the ridge segments E2 and E9, which lack the characteristic alvinocarid shrimps, bathymodiolid mussels and siboglinid worms found at Atlantic, Indian and Pacific hydrothermal vents, respectively [21]. Instead, biomass at the ESR vents is dominated by anomuran crabs (*Kiwa* sp.), stalked barnacles (*Vulcanolepas* sp.) and large peltospiroid gastropods [22], indicating a new biogeographic province [21]. Furthermore, there are differences in the end-member vent fluid chemistry between the E2 and E9 vent fields as well as within field between northern (E9N) and southern (E9S) areas of E9 [21].

Stable isotopes of carbon (^13^C/^12^C expressed as δ^13^C), nitrogen (^15^N/^14^N expressed as δ^15^N) and sulfur (^34^S/^32^S expressed as δ^34^S) have been used to examine hydrothermal vent community trophodynamics [23], [24]. δ^13^C can be used to characterise the various carbon sources utilised by vent macroconsumers [25]. This is done by comparing the expected carbon fractionation between dissolved inorganic carbon (DIC) and the macroconsumer’s tissue. Enzymatic reactions catalysed by the ribulose-1,5-biphosphate carboxylase/oxygenase form I (RuBisCO form I) of the CBB cycle (22‰ to 30‰: [26], [27], [28]) exhibit greater fractionation than those of the rTCA cycle (2‰ to 14‰: [29], [30], [31]). Once organic material is incorporated into the macroconsumer food web, carbon trophic discrimination (Δ^13^C) is small, ranging from 0 to 1.5‰ between the food source and consumer [32]. δ^34^S also identifies energy sources (sulfur trophic discrimination, −1‰ to 2‰: [32]). The large difference in δ^34^S between seawater sulphate and sulfides at hydrothermal vents [33] results in organic matter of photosynthetic (∼16‰ to 19‰) and chemosynthetic (−9‰ to 10‰) origin having distinctive δ^34^S values [34], [35]. The greater trophic discrimination (2‰ to 5‰) in δ^15^N between consumer and food source provides information on the trophic position of an organism relative to a primary consumer [32]. Therefore, the isotopic value of a vent macroconsumer is the product of the following factors: (1) the inorganic substrate and its isotopic value used by the chemoautotroph; (2) the isotopic discrimination processes occurring during metabolic reactions involving inorganic substrates to create organic compounds (e.g. CBB or rTCA cycles) by the chemoautotroph; (3) food source-macroconsumer trophic interactions (e.g. endosymbiont-host, predator-prey) that occur as a function of (1) and (2); and (4) the physiology associated with the macroconsumer’s isotopic trophic discrimination.

The goal of the present research was to investigate intra- and inter-site patterns in the trophic assemblages of macroconsumers occurring at hydrothermal vents on the ESR using δ^13^C, δ^34^S and δ^15^N. Specifically, the aims were to: (1) compare δ^13^C_DIC_ among vent sites and thus establish difference in the isotopic inorganic substrates used by chemoautotrophs; (2) compare δ^13^C, δ^15^N and δ^34^S between vent and benthic non-vent fauna to assess any photosynthetic inputs into the hydrothermal vent food web; (3) investigate differences in trophic structures among the three sites; and (4) assess which species are driving any differences in trophic structure. The investigation provides a unique opportunity to examine differences in trophic structure at the scale of tens of metres to 100s of kilometres in a newly discovered hydrothermal vent biogeographical province.

## Materials and Methods

### Ethics Statement

Permits for the fieldwork were granted by the United Kingdom Foreign and Commonwealth Office. This study met the ethical requirements of the affiliated research institutions for research utilising animal tissues. No animal husbandry or laboratory controlled experiments were part of the research that required permits from the UK Home Office. The fish were collected at a water depth of 2500 m, which meant that they were dead when they arrived on deck as a result of changes in pressure. This was the case with the majority of the animals dissected within this study. The research also adhered to the Inter Ridge code of conduct for sampling hydrothermal vents (http://www.interridge.org/IRStatement).

### Study Sites

**Figure 1:**
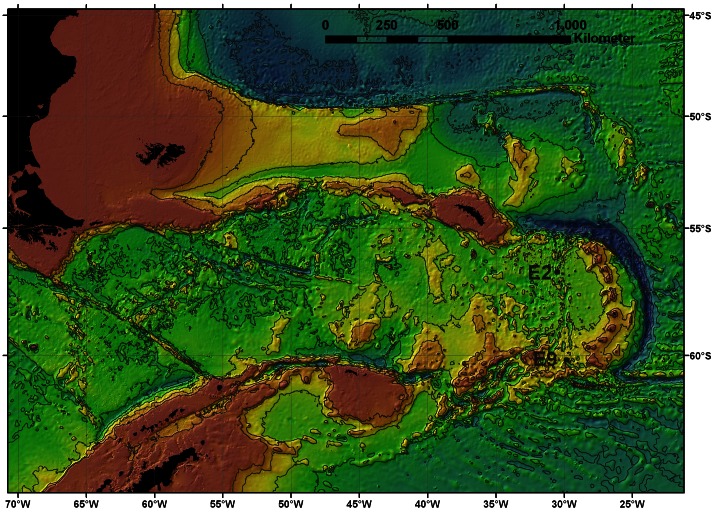
Bathymetric map illustrating positions of the E2 and E9 vent sites (black circles). The vent sites are located at the northern and southern ends of the East Scotia Ridge (ESR), located in the Atlantic sector of the Southern Ocean. The map shows the position of the ESR in relation to South America and the Antarctic Peninsula.

The E2 and E9 vent fields are situated approximately 440 km apart at 56° 05.35′S, 30° 19.20′W and 60° 02.50′ S, 29° 58.93′ W, respectively (Fig. 1). E2 is at a depth of ∼2600 m and seafloor topography is complex with a series of terraced features and lobed pillow basalts filling a major north-south steep-sided fissure [21]. The main high-temperature and diffusive venting occurred at an intersection between this fissure and an east-west running fault or scarp [21]. E9 was located at ∼2400 m depth and its topography was relatively flat with sheet lava, a series of lava drain back features and collapsed pillow basalts. A series of north-south fissures were found with venting mainly occurring on the most western [21], [22]. The end-member fluid chemistry exiting chimneys differed between the northern and southern sections of E9 [21], therefore E9N and E9S are here considered to be separate sites. Ambient seabed water temperatures were 0.0°C at E2 and between −0.1°C and −1.3°C at E9 [21].

### Sample Collection and Ship-board Processing

Samples were collected onboard the R.R.S *James Cook* during the 2010 austral summer (7 January to 21 February) using the remotely operated vehicle (ROV) *Isis*. High temperature and diffuse flow fluids were collected for DIC using titanium samplers, equipped with an inductively coupled link high temperature sensor. The nozzle of the titanium sampler was inserted into the chimney orifice for high temperature fluid samples and once the temperature reading became stable the fluid was collected. For diffuse flow samples, a circular titanium housing was placed over the area of diffuse venting to minimise the entrainment of seawater. Once the diffuse flow was visible exiting the top of the housing, the titanium sampler was inserted into the opening and the diffuse flow sample was collected once the temperature reading was stable. On board, an aliquot for stable isotope analysis of DIC was sampled to exclude air and poisoned with mercury chloride.

Vent macroconsumers were collected by suction sampler or scoop with species separated into a series of acrylic chambers or perspex boxes to avoid predation or contamination. Six species were collected at all three sites. No female *Kiwa* sp. were collected from E9N or E9S. Fish and pycnogonids were collected using large collapsible and small metal baited traps deployed from the ROV. Non-vent macroconsumers were collected from metres to tens of metres away from active venting where there were no obvious signs of hydrothermal influence, i.e. no bacterial mat, and where temperature was consistent with local Antarctic bottom water. Non-vent samples were collected on separate dives from those for vent fauna to avoid contamination. Only one non-vent species was collected from E2 and sampling was limited to the areas adjacent to E9N because of ROV operational time constraints. Potential food sources were collected by scraping material from rocks collected by ROV manipulators and epibionts from the ventral setae of the decapod *Kiwa* sp. Particulate suspended material was collected from the acrylic chambers, which was sampled incidentally during faunal collection. Samples were sorted on board to the lowest possible taxonomic resolution. The majority of the vent species are undescribed to date.

Faunal samples were frozen at −80°C whole or after dissection, depending on their size, for stable isotope analysis. Muscle was removed from the chelipeds of *Kiwa* sp., foot dissected from Peltospiroidea sp., tube feet removed from the asteroids Stichasteridae sp. and *Freyella* cf *fragilissima* and tentacles removed from the anemones. Legs were removed from the pycnogonids *Colossendeis* cf. *concedis* and *C.* cf. *elephantis*, while *Sericosura* spp. was sampled whole. The gastropods Provannidae sp. 1 and 2, *Lepetodrilus* sp., and juvenile Peltospiroidea sp. (<7 mm shell length), and the stalked barnacle *Vulcanolepas* sp. were removed from their shells and sampled whole. White muscle tissue was dissected from the anterior dorso-lateral region of the zoarcid fish.

### Sample Processing Onshore

Each end-member and diffuse flow DIC sample was prepared for isotopic analysis by removing a 1 mL water sample and transferring it into a separate vial. The headspace was flushed with helium, phosphoric acid was injected into the vial and then the contents were vortex mixed. The samples were then left to react for 24 hours to ensure complete conversion of all DIC to CO_2_ for isotopic analysis. The CO_2_ was then analysed by continuous-flow isotope ratio mass spectrometry (IRMS) using a Europa Scientific 20–20 IRMS by Iso-Analytical (Crewe, United Kingdom). Samples were run in duplicate and the mean is reported. An internal reference gas (IA-R060, δ^13^C = −36.08‰ ± SD 0.13) was used to determine the δ^13^C_DIC_ values and is traceable to the International Atomic Energy Agency standard, NBS-19. Concentrations of CH_4_ in the water samples were insufficient for isotope analysis.

Faunal tissue samples were freeze dried and ground to a homogenous powder using a pestle and mortar. Aliquots of fauna, particulate suspended material and material scraped from rocks were tested for carbonates prior to analysis with 0.1 N HCl. If the sample effervesced, this indicated carbonates were present and it was subsequently acidified by further addition of HCl until the effervescence ceased. Samples were re-dried at 50°C for 48 hours. If the sample did not effervesce, no acidification was carried out. Aliquots for δ^13^C analysis were not lipid extracted. Any confounding lipid effects due to metabolic processes would not affect the interpretation of the ultimate carbon sources of the vent fauna described by δ^13^C because of the large differences in the δ^13^C values of trophic end-members.

Approximately 0.7 mg of powder was weighed into a tin capsule for carbon and nitrogen IRMS. For sulfur, 2 mg of sample and 4 mg of the catalyst vanadium pentoxide were weighed into each tin capsule. Dual stable carbon and nitrogen isotope ratios were measured by continuous-flow IRMS using a Costech Elemental Analyser interfaced with Thermo Finnigan Delta Plus XP (Natural Environment Research Council, Life Sciences Mass Spectrometry Facility, SUERC, East Kilbride, United Kingdom). Two laboratory standards were analysed for every ten samples in each analytical sequence. These alternated between paired alanine standards, differing in δ^13^C and δ^15^N, and an internal laboratory gelatin standard. Sulfur was analysed by Iso-Analytical using a SERCON Elemental Analyser coupled to a Europa Scientific 20–20 IRMS. Laboratory standards of barium sulphate (two sets of differing δ^34^S) and silver sulfide were used for calibration and drift correction. An internal standard of whale baleen was used for quality control (n = 28, 16.34‰ ± SD 0.21). Stable isotope ratios were expressed in delta (δ) notation as parts per thousand/permil (‰). All internal standards are traceable to the following international standards: v-PDB (Pee Dee Belemnite), AIR (atmospheric nitrogen) and NBS-127 (barium sulphate), IAEA-S-1 (silver sulfide) and IAEA-SO-5 (barium sulphate). An external reference material of freeze dried and ground deep-sea fish white muscle (*Antimora rostrata*) was also analysed (δ^13^C, n = 24, −18.94‰ ± SD 0.09; δ^15^N, n = 24, 13.11‰ ± SD 0.38; δ^34^S, n = 30, 18.20‰, ± SD 0.59).

### Data Analysis

Data were assessed for normality using a Shapiro-Wilk test before statistical tests examining spatial patterns in trophic structure and species stable isotope values. Homogeneity, or otherwise, of variances is ecologically informative, for example in identifying distinct energy sources at the base of the food web [36]. Inter-site differences in trophic structure were examined using a Fligner-Killeen test for homogeneity of variance to assess differences in the spread of the mean stable isotope values of each species. Inter-site differences in species were analysed using a one-way ANOVA followed by Tukey’s honest significant difference (HSD) when variance was homogeneous among sites. Welch’s ANOVA followed by t-tests were used when there was heterogeneity of variance among sites because it uses adjusted degrees of freedom to protect against Type I errors when variances are unequal [37]. A Bonferroni correction (p = 0.05/*n*) was used for multiple comparisons. When data were not normally distributed, a two sample Wilcoxon test was used. All statistics were preformed in R version 12.13.1 [38].

## Results

### Dissolved Inorganic Carbon Stable Isotope Values

Mean (± SD) δ^13^C_DIC_ of high temperature and diffuse flow fluids are summarised in Table 1. δ^13^C_DIC_ of high temperature samples collected at two E2 locations were −4.7‰ (±0.0) (max temperature 351.0°C) and −2.5‰ (±0.1) (max temperature 323.0°C). At E9N, δ^13^C_DIC_ from separate orifices of the same chimney structure were −4.6‰ (±0.0) (max temperature 380.2°C) and −4.5‰ (±0.0) (max temperature 357.0°C). No high temperature fluids were collected from E9S for δ^13^C_DIC_ analysis because the pressure was too high within the titanium samples to safely and accurately collect a representative sample. Diffuse flow samples from amongst *Kiwa* sp. and anemones, at E2, had δ^13^C_DIC_ values of 0.8‰ (±0.1) (max temperature 19.9°C) and 0.2‰ (±0.2) (max temperature 3.5°C), respectively. A single diffuse flow sample collected from amongst an aggregation of *Kiwa* sp. at E9N had a δ^13^C_DIC_ value of 1.5‰ (±0.1) (max temperature 12.6°C). At E9S, diffuse flow samples from amongst *Kiwa* sp. had a δ^13^C_DIC_ value of 0.9‰ (±0.1) (max temperature 19.9°C), while a sample taken from a mixed aggregation of *Kiwa* sp. and peltospiroid gastropods had δ^13^C_DIC_ value of 0.1‰ (±0.1) (max temperature 5.0°C).

**Table 1:** δ^13^C values of dissolved inorganic carbon (DIC) sampled from high temperature and diffuse flow venting from the E2 and E9 ridge segments of the East Scotia Ridge, Southern Ocean.

Site	Temperature (°C)	δ^13^C DIC
E2	351.0	−4.7 (0.0)
	323.0	−2.5 (0.1)
	19.9	0.8 (0.1)
	3.5	0.2 (0.2)
E9N	380.2	−4.7 (0.0)
	357.0	−4.7 (0.0)
	12.6	1.5 (0.1)
E9S	19.9	0.9 (0.1)
	5.0	0.1 (0.1)

Standard deviations are in parentheses.

### Comparison between Vent and Benthic Non-vent Macro-consumers at E9N

**Figure 2:**
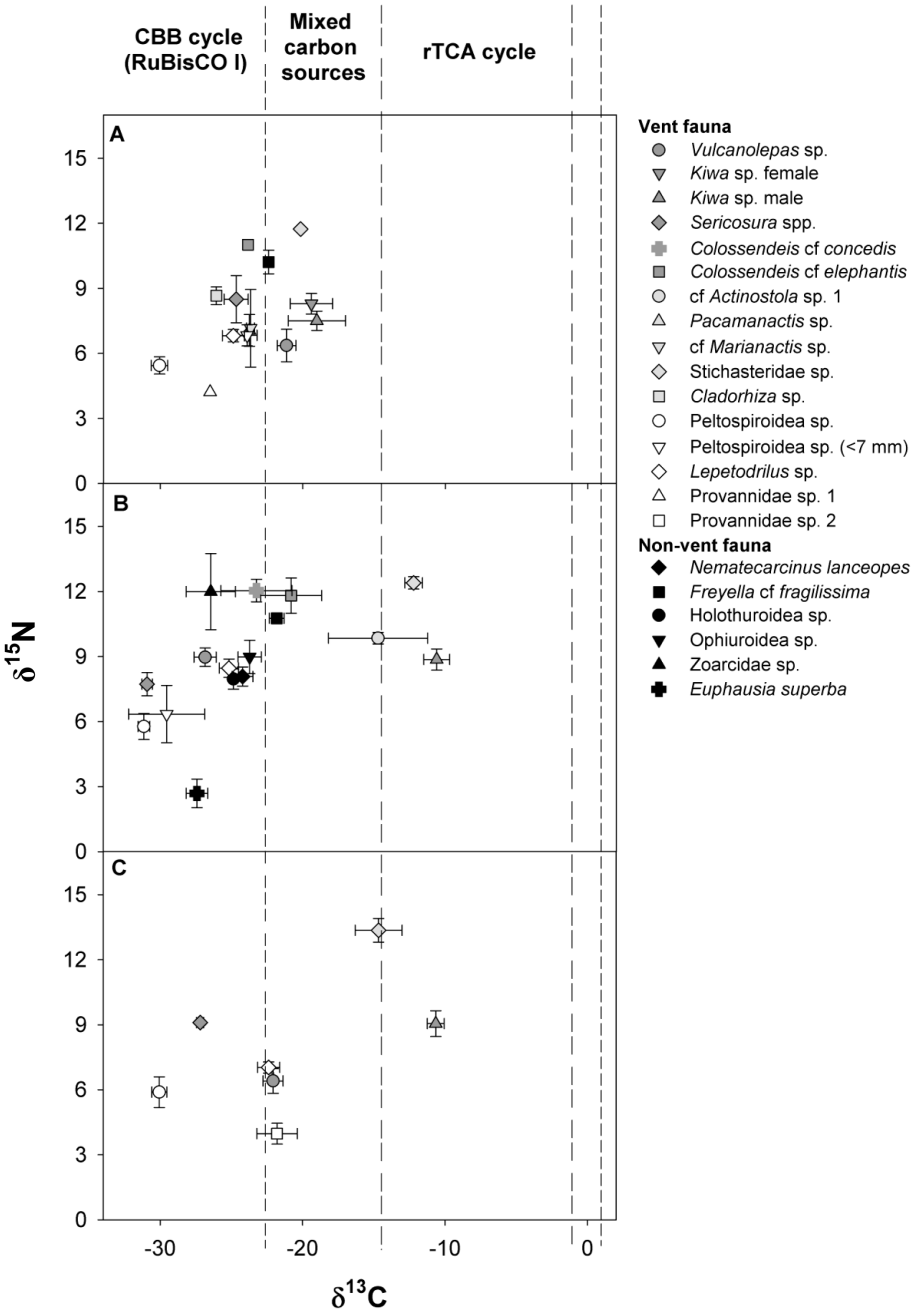
δ^13^C and δ^15^N values of macroconsumers collected from the East Scotia Ridge, Southern Ocean. The values represent means (± standard deviations) for hydrothermal vent and non-vent macroconsumers from the three sample sites: (a) E2, (b) E9N and (c) E9S. Dashed vertical lines represent potential ranges of δ^13^C values indicative of carbon sources sustaining macroconsumers at the ESR: triple dashed line represents the Calvin-Benson-Bassham (CBB) cycle utilising form I RuBisCO, double dashed line represents the reductive tricarboxylic acid (rTCA) cycle, mixed carbon sources occur between the triple and double dashed line and the continuous dashed line represents the approximate δ^13^C values of the dissolved inorganic carbon from the diffuse flow areas.

**Figure 3:**
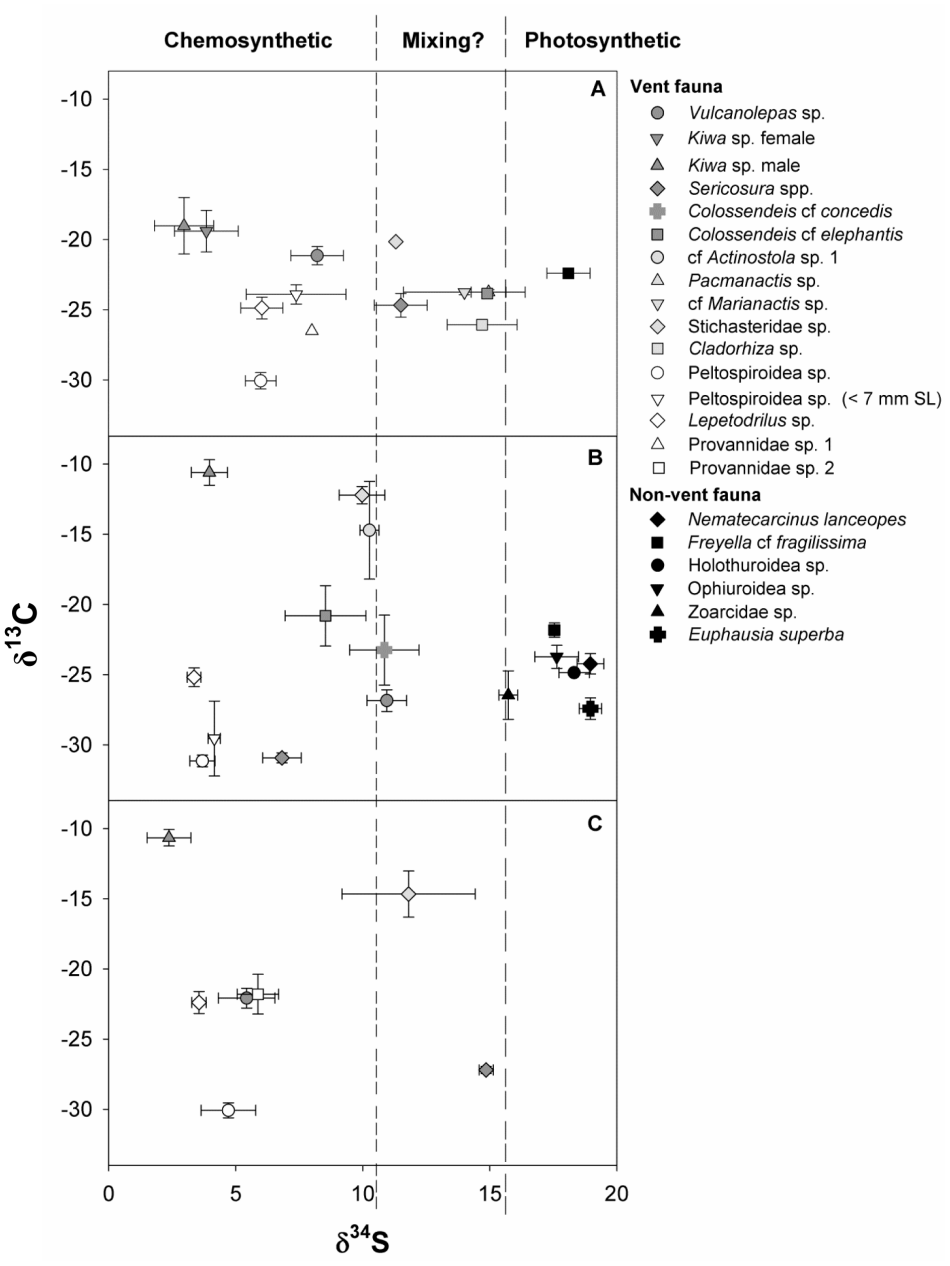
δ^13^C and δ^34^S values of macroconsumers collected from the East Scotia Ridge, Southern Ocean. The values represent means (± standard deviations) for hydrothermal vent and non-vent macroconsumers from the three sample sites: (a) E2, (b) E9N and (c) E9S. δ^34^S values between the triple and double dashed lines represent potential areas of isotopic mixing between chemosynthetic and photosynthetic food sources.

At E9N, mean δ^13^C and δ^15^N values of vent fauna overlapped with non-vent benthic fauna (Welch’s t-test, δ^13^C DF = 10.59, t = 0.66, p = 0.52; Welch’s t-test, δ^15^N DF = 10.42, t = −0.30, p = 0.76; Fig. 2, Tables 2 & 3) while mean δ^34^S values differed between non-vent benthic fauna and vent fauna (Welch’s t-test, DF = 12.56, t = −9.08, p<0.01) (Fig. 3, Tables 2 & 3).

**Table 2:** Mean δ^13^C, δ^15^N and δ^34^S values (‰) of non-vent deep-sea fauna collected from the E2 and E9 ridge segments of the East Scotia Ridge, Southern Ocean. Standard deviations are in parentheses.

Taxonomic group	Species	Site	N	δ^13^C	δ^ 34^S	δ^ 15^N
**Crustacea**						
Decapoda	*Nematocarcinus lanceopes*	E9	5	−24.2 (0.7)	18.9 (0.5)	8.1 (0.4)
	*Euphausia superba*	E9	3	−27.4 (0.8)	19.0 (0.1)	2.7 (0.7)
**Echinodermata**						
Asteroidea	*Freyella* cf *fragilissima*	E2	3	−22.4 (0.3)	18.0 (0.8)	10.2 (0.6)
	*Freyella* cf *fragilissima*	E9	2	−21.8 (0.5)	17.5 (0.2)	10.8 (0.2)
Holothuroidea	Holothuroidea sp.	E9	3	−24.9 (0.0)	18.3 (0.6)	8.0 (0.4)
Ophiuroidea	Ophiuroidea sp.	E9	3	−23.7 (0.8)	17.6 (0.9)	9.0 (0.8)
**Vertebrata**						
Osteichthys	Zoarcidae sp.	E9	4	−26.5 (1.7)	15.7 (0.4)	12.0 (1.8)

**Table 3:** Mean δ^13^C, δ^15^N and δ^34^S values (‰)of hydrothermal vent fauna collected from the E2 and E9 ridge segments of the East Scotia Ridge, Southern Ocean.

Taxonomic group	E2				E9N				E9S			
	N	δ^13^C	δ^34^S	δ^15^N	N	δ^13^C	δ^34^S	δ^15^N	N	δ^13^C	δ^34^S	δ^15^N
**Cirripedia**												
*Vulcanolepas* sp.	22	−21.1 (0.6)	8.2 (1.0)	6.3 (0.7)	23	−26.9 (0.8)	11.0 (0.8)	9.0 (0.4)	23	−22.1 (0.8)	5.4 (1.1)	6.4 (0.6)
**Decapoda**												
*Kiwa* sp. female	20	−19.4 (1.5)	3.9 (1.3)	8.2 (0.5)	0	–	–	–	0	–	–	–
*Kiwa* sp. Male	18	−19.0 (2.0)	3.0 (1.2)	7.5 (0.5)	22	−10.6 (0.9)	4.0 (0.7)	8.9 (0.5)	30	−10.7 (0.6)	2.4 (0.9)	9.1 (0.6)
**Pycnogonida**												
*Sericosura* spp.	6	−24.7 (0.9)	11.9 (0.4)	8.5 (1.3)	9	−30.9 (0.5)	6.8 (0.8)	7.7 (0.5)	2	−27.2 (0.3)	14.9 (0.3)	9.1 (0.1)
*Colossendeis* cf *concedis*	0	–	–	–	6	−23.3 (2.5)	10.9 (1.4)	12.1 (0.5)	0	–	–	–
*Colossendeis* cf *elephantis*	1	−23.8	14.9	11.0	3	−20.8 (2.2)	8.5 (1.6)	11.8 (0.8)	0	–	–	–
**Anthozoa**												
cf *Actinostola* sp. 1	0	–	–	–	4	−14.7 (3.5)	10.3 (0.4)	9.9 (0.3)	0	–	–	–
*Pacmanactis* sp.	5	−23.8 (0.2)	14.9 (0.7)	7.1 (0.7)	0	–	–	–	0	–	–	–
cf *Marianactis* sp.	5	−23.7 (0.3)	14.0 (2.4)	7.2 (1.8)	0	–	–	–	0	–	–	–
**Asteroidea**												
Stichasteridae sp.	1	−20.2	11.3	12.3	5	−12.2 (0.6)	10.0 (0.9)	12.4 (0.4)	5	−14.7 (1.6)	11.8 (2.6)	13.4 (0.6)
**Gastropoda**												
Peltospiroidea sp.	19	−30.1 (0.6)	6.0 (0.6)	5.4 (0.4)	22	−31.2 (0.4)	3.7 (0.5)	5.8 (0.6)	15	−30.1 (0.5)	4.7 (1.1)	5.9 (0.7)
Peltospiroidea sp (<7 mm)	4	−23.9 (0.7)	7.4 (2.0)	6.8 (0.5)	5	−29.6 (2.7)	4.2 (0.2)	6.4 (1.3)	0	–	–	–
Provannidae sp. 1	1	−26.5	8.0	4.2	0	–	–	–	0	–	–	–
P–rovannidae sp. 2	0	–	–	–	0	–	–	–	4	−21.8 (1.4)	5.9 (0.8)	4.0 (0.5)
*Lepetodrilus* sp.	5	−24.9 (0.8)	6.4 (0.5)	6.8 (0.3)	4	−25.2 (0.7)	3.4 (0.3)	8.5 (0.4)	4	−22.4 (0.8)	3.6 (0.3)	7.0 (0.3)
**Cladorhizidae**												
*Cladorhiza* sp.	5	−26.1 (0.4)	14.7 (1.4)	8.7 (0.4)	0	–	–	–	0	–	–	–
**Potential food sources**												
Particulate suspended material	3	−23.2 (5.4)	10.0 (1.1)	−0.1 (4.9)	0	–	–	–	0	–	–	–
Rock scrapings	0	–	–	–	1	−23.2	0.8	2.4	1	−31.1	–	1.9
*K*–*iwa* n. sp episymbiont	5	−18.9 (5.3)	7.5 (0.3)	3.3 (1.5)	0	–	–	–	5	−9.9 (0.3)	6.6 (0.2)	5.2 (0.8)

Standard deviations are in parentheses and - indicates no data.

### Intra- and Inter-site Differences in Community Trophodynamics

Eleven, ten and seven species were collected at E2, E9N and E9S respectively for stable isotope analysis (Table 3). The ranges of mean δ^13^C values of the vent fauna differed amongst the three sites (Fligner-Killeen test, DF = 2, χ^2^ = 6.46, p<0.05). E2 had the narrowest δ^13^C range (−29.9‰ to −19.0‰), whereas at E9N and E9S δ^13^C ranged from −31.4‰ to −9.9‰ and −30.0‰ to −10.5‰, respectively (Fig. 2). Across the three sites Peltospiroidea sp. had the lowest values while *Kiwa* sp. had the highest δ^13^C values (Fig. 2, Table 3), and *Lepetodrilus* sp., *Vulcanolepas* sp., *Pacmanactis* sp. and *Colossendeis* spp. all had intermediate δ^13^C values (Fig. 2, Table 3). However, there was no overall difference in mean δ^13^C values among sites for the combined data across species (Welch’s ANOVA, DF = 2.00, F = 0.59, p = 0.56). The range and mean δ^34^S values (Fig. 3, Table 3) did not differ among sites (Fligner-Killeen test, DF = 2, χ^2^ = 0.84, p = 0.65; ANOVA, DF = 2, 26, F = 1.94, p = 0.16), however *Kiwa* sp. had the lowest δ^34^S at E2 and E9S while *Lepetodrilus* sp. had the lowest δ^34^S values at E9N (Fig. 3, Table 3). The highest vent fauna δ^34^S values were in *Pacmanactis* sp. (E2), *Vulcanolepas* sp. (E9N) and *Sericosura* spp. (E9S) (Fig. 3, Table 3). Neither the range nor the mean δ^15^N values differed among sites (Fligner-Killeen test, DF = 2, χ^2^ = 0.40, p = 0.83; ANOVA, DF = 2, 26, F = 1.19, p = 0.31). The provannid gastropods at E2 and E9S had the lowest δ^15^N values while Peltospiroidea sp. had the lowest values at E9N (Fig. 2, Table 3). The stichasterid sp. consistently had the highest δ^15^N values relative to the other vent fauna at each site (Fig. 2, Table 3).

### Spatial Differences in Macroconsumer Trophodynamics

*Vulcanolepas* sp. exhibited spatial differences in δ^13^C, δ^15^N and δ^34^S but there was no consistent pattern in isotopic differences among sites (Table 4). Male and female *Kiwa* sp. at E2 did not differ in δ^13^C but males were lower in δ^15^N and δ^34^S than females (Table 5). Male *Kiwa* sp. showed spatial differences in each stable isotope (Table 4). δ^13^C of the males showed a greater range (Fligner-Killeen test, DF = 2, χ^2^ = 10.91, p<0.01) and lower values at E2 than E9N and E9S (Table 3 & 4). The epibionts attached to the ventral surface of male *Kiwa* sp. also exhibited a greater spread of δ^13^C values at E2 than E9S (F-test, DF = 4, 3, F = 244.46, p<0.01) as well as lower δ^13^C but higher δ^15^N values at E2 than E9S (Table 3 & 5). *Sericosura* spp. δ^13^C and δ^34^S values varied amongst sites but δ^15^N values scarcely did (Table 4). δ^13^C and δ^34^S values were lowest at E9N but highest at E2 for δ^13^C and E9S for δ^34^S (Table 3). Peltospiroidea sp. showed spatial differences in δ^13^C and δ^34^S but not in δ^15^N (Table 4). δ^34^S values differed among all sites but E9N δ^13^C values were lower than those at E9S and E2 (Table 4). Stichasteridae sp. revealed differences between all sites for δ^13^C and δ^15^N but for δ^34^S only between E2 and E9N (Table 5).

**Table 4:** Results of ANOVA and *post-hoc* Tukey honest significant differences tests for the differences in stable isotope values of vent fauna among the three sites on the East Scotia Ridge.

Species	δ^13^C				δ^ 34^S				δ^15^N			
	DF	F	p	*Post-hoc*	DF	F	p	*Post-hoc*	F	DF	p	*Post-hoc*
*Vulcanolepas* sp.	2, 63	403.18	<0.01	E9N<E9S<E2	2, 63	176.16	<0.01	E9S<E2 = E9S	2, 63	138.26	<0.01	E2 = E9S<E9N
*Kiwa* sp. male	2, 31.36	147.29	<0.01	E2< E9S = E9N*	2, 66	19.52	<0.01	E2 = E9S<E9N	2, 66	52.64	<0.01	E2< E9N = E9S
*Sericosura* spp.	2, 15	215.00	<0.01	E9N<E9S<E2	2, 15	100.61	<0.01	E9N<E2< E9S	2, 15	3.39	0.06	NA
Peltospiroidea sp.	2, 52	29.50	<0.01	E9N<E9S = E2	2, 52	49.26	<0.01	E9N<E9S<E2	2, 52	2.90	0.06	NA
*Lepetodrilus* sp.	2, 10	17.41	<0.01	E2 = E9N<E9S	2, 10	31.99	<0.01	E9N = E9S<E2	2, 10	32.10	<0.01	E2 = E9S<E9N

* Welch’s ANOVA with *post hoc* analysis by t-test with Bonferroni correction (p = 0.05/3 = 0.017).

**Table 5:** Results of t-tests for between-sites differences in stable isotope values of vent fauna at the East Scotia Ridge.

Species	Comparison	δ^13^C			δ^ 34^S			δ^ 15^N		
		DF	t	p	DF	t	p	DF	t	p
*Kiwa* sp.	E2 female v male	36	−0.50	0.62	36	2.23	<0.05	36	5.13	<0.01
*Kiwa* sp. Epibionts	E2 v E9S	4.05	−3.81	<0.05*	6.93	4.57	<0.01*	2	na	<0.05Ψ
Stichasteridae sp.	E2 v E9N	4	28.92	<0.01	4	−3.29	<0.05	4	5.14	<0.01
	E2 v E9S	4	7.28	<0.01	4	0.385	0.59	4	5.94	<0.01
	E9N v E9S	8	3.64	<0.01	8	−1.76	0.11	8	−3.49	<0.05
*Colossendeis* cf *elphantis*	E2 v E9N	2	2.45	0.13	2	−6.93	<0.05	2	1.71	0.23

* Welch’s t-test,

Ψ Wilcoxon test.

## Discussion

This study described the trophic structure of a new vent biogeographical province recently discovered on the ESR in the Southern Ocean [21]. In addressing this aim, the study shared the challenges of preceding work in characterising energy sources, separating isotopic overlap and mixing of energy sources, and following energy sources into subsequent predator-prey relationships. However, the tri-isotope approach and integration of both vent chemistry and microbiology, here, provided a more holistic understanding of vent trophic ecology at within- and among-vent field scales.

### Intra-site Trophic Interactions and Energy Sources

Scarcity of Δ^13^C estimates between inorganic carbon and cellular biomass for primary producers at hydrothermal vents [27], [30] makes interpretation of the origin of organic carbon fixed within the hydrothermal vent system and assimilated by macroconsumers tentative for species not within a symbiotic or known predator-prey relationship. Diffuse flow δ^13^C_DIC_ of approximately 1‰ at the ESR vent fields suggests ESR vent macroconsumers with δ^13^C values <−22‰ are assimilating carbon fixed via the CBB cycle because the net fractionation associated with fixing inorganic into organic carbon for RuBisCO form I ranges from −22‰ to −30‰ [26], [27]. Peltospiroidea sp. housed an endosymbiotic gamma-Proteobacteria (K. Zwirglmaier unpublished data) and is within the δ^13^C range expected for carbon fixed via RuBisCO form I at all three locations. Molluscs containing a single strain of endosymbiotic gamma-Proteobacteria living in other biogeographical vent provinces include some species of bathymodiolid mussels, vesicomyid clams and *Ifremeria* gastropods, all of which have δ^13^C values between −37‰ and −27‰ [39], [40]. Other species of ESR vent macroconsumers, which had δ^13^C values <−22‰ included *Vulcanolepas* sp. (E2 and E9S), *Sericusora* spp., E2 anemones and *Lepetodrilus* sp. These species consume free-living bacteria [14], [23] so organic carbon fixed via other carbon fixation pathways cannot be ruled out as part of their assimilated diet.

Vent macroconsumers inhabiting the hottest areas of the hydrothermal vent tolerable to metazoan life, including rimicarid shrimps, polychaetes *Alvinella* spp. and *Riftia pachyptila* and some alvinoconchid gastropods, tend to assimilate rTCA-fixed carbon from their diet [11], [41], [42] and have δ^13^C values >−16‰ [14], [25], [43], [44]. As δ^13^C_DIC_ is approximately 1‰ at the ESR sites, vent macroconsumers utilising carbon fixed via the rTCA cycle would have had δ^13^C values >−13‰; assuming a −2‰ to −14‰ net fractionation between the inorganic substrate and organic product catalysed by the enzymes involved in the rTCA cycle [29], [30]. *Kiwa* sp. living at E9N and E9S, along with its epibionts, had δ^13^C values that were >−12‰ and are found in areas close to discharging vent fluids [22]. This potentially indicates the epibionts living on *Kiwa* sp. ventral setae were fixing carbon via the rTCA cycle. *Kiwa* sp. was also ^15^N-enriched by between 3.8‰ and 4.2‰ relative to its epibionts, suggesting the epibionts were an important food source. A similar episymbiotic relationship between the ESR kiwid is therefore hypothesised to that of *Kiwa puravida*, for which lipid, stable isotope and behavioural analyses indicate the harvesting of epibiont bacteria [45]. Stichasteridae sp. (∼−13‰) and cf *Actinostola* sp. (∼−14‰) also appeared to be assimilating carbon indicative of the rTCA cycle at E9N and E9S.

Several vent macroconsumers fell within the range of δ^13^C values indicative of mixed carbon sources. Those within the δ^13^C −22‰ to −15‰ range may consume free-living bacteria or are predators or scavengers that utilise a number of trophic pathways. At the ESR hydrothermal vents, *Lepetodrilus* sp., Provannidae sp. 2, *Vulcanolepas* sp., *Kiwa* sp., Stichasteridae sp. and *Colossendeis* cf. *elephantis* fell into this range at one or more sites. Related species of *Lepetodrilus* sp., Provannidae sp. 2 and *Vulcanolepas* sp. are all thought to consume free-living bacteria at other vents sites [14], [23]. Such feeding can result in consuming heterogeneous bacterial communities, which have multiple pathways for carbon fixation and elemental cycling [9], [46], [47]. The biological cycling of carbon is very complex at hydrothermal vents because of the multiple single carbon substrates for carbon fixation (e.g. CO_2_, CH_4_, CO), spatial variability in the δ^13^C value of the substrate and various microbial primary producers associated with different carbon fixation pathways [7], [11], [48]. Furthermore, the incorporation of photosynthetic derived carbon as particulate or dissolved organic matter is possible and may provide some nutrition to vent macroconsumers [12], [13]. Therefore, complex isotopic mixes of food sources are available to these species.

The majority of ESR vent macroconsumers had δ^34^S values less than or equal to the 10‰ threshold, indicating chemosynthetic food sources [49]. Species exceeding the 10‰ value occurred mainly at E2 in the anemones *Pacamanactis* sp. and cf *Marianactis* sp, the sponge *Cladorhiza* sp., the pycnogonids *C. elephantis* and the stichasterid seastar along with *Sericosura* spp. and stichasterid seastar at E9S. All had δ^34^S values between 10‰ and 16‰. Mixing of epipelagic photosynthetic and hydrothermal vent chemosynthetic production sources at these sites cannot be ruled out.

Determining intra-site differences in food sources and trophic interactions using δ^34^S is challenging for macroconsumers with δ^34^S values <10‰ because the δ^34^S values of inorganic substrates and the net fractionation effect between inorganic substrates and products for primary producers and consumers are uncertain. At E9, δ^34^S appeared to increase from macroconsumers living closest to vent openings and within diffuse flow areas (i.e. *Kiwa* sp., Peltospiroidea sp. and *Lepetodrilus* sp.) to those in the periphery (i.e. anemones, stichasterid seastars and *Colossendeis* spp.). It is unclear why an increase in δ^34^S occurred from the centre of the vent to the periphery: it may be the result of changes in sulfide speciation [50] or other sulfur sources with increasing distance from the vent opening [33], differences in levels of sulfide exposure [50], incorporation of epipelagic photosynthetic primary production or a combination of the above.

Stichasterid seastars, cf *Actinostola* sp. and *Colossendeis* spp. consistently had the highest δ^15^N values of all the ESR vent macroconsumers, which suggested they occupied the highest trophic positions of those predators sampled. Behavioural observations [22] and δ^13^C values indicated that *Kiwa* sp. is consumed by stichasterid seastar and cf *Actinostola* sp. 1 but only the stichasterid seastar had δ^15^N values indicative of a higher trophic position than *Kiwa* sp. In the case of *Colossendeis* spp., feeding on anemones occurs at the ESR vent sites [22] and at E2 all three stable isotopes indicated a strong predator-prey link. At E9N there was a large difference in δ^13^C and δ^34^S between cf *Actinostola* sp. 1 and the two species of *Colossendeis* as well as lower δ^15^N in these pycnogonids compared to cf *Actinostola* sp. 1. This suggests that at E9N the feeding incidents between cf *Actinostola* sp. 1 and *Colossendeis* spp. are either rare or stable isotope values of *Colossendeis* spp. are strongly affected by isotopic mixing of different energy sources (δ^13^C and δ^34^S) and feeding over multiple trophic positions (δ^15^N).

It is evident from the ESR hydrothermal vent food webs that predators may have similar or lower δ^15^N values than their prey. Calculating trophic position assuming taxon specific nitrogen trophic discrimination factors [23] or applying the more universal value of 3.4‰ [12] was not undertaken within this study because they may have provided erroneous results. Establishing a suitable δ^15^N baseline is problematic because: the macroconsumer with the lowest δ^15^N differed among locations, is confounded by the use of different tissues (e.g. whole animals, muscle) to construct the food webs [32] and the observed high δ^15^N variability in potential food sources. Compound-specific amino acid stable isotope analysis may provide higher resolution information on the organic nitrogen compounds assimilated by vent macroconsumers because the isotopic values of different amino acids record trophic and basal source information [51], [52]. Thus it may circumvent some of the limitations of bulk δ^15^N analysis and provide a better understanding of nitrogen cycling at hydrothermal vents.

### Spatial Patterns in Macroconsumer Trophodynamics

Large spatial differences in δ^13^C values for *Kiwa* sp., Stichasteridae sp. and *Sericosura* spp. were attributed primarily to differences in carbon fixation pathways at the base of the food web, which is in turn transferred to higher trophic positions. δ^13^C values of *Kiwa* sp. differed by ∼9‰ between E2 and E9S as did that of associated *Kiwa* sp. epibionts. Also, epsilon-Proteobacteria dominated the epibiont community at E9 with gamma-Proteobacteria largely absent, compared to a mix of gamma- and epsilon-Proteobacteria at E2 (K. Zwirglmaier unpublished data). All epsilon-Proteobacteria to date use the rTCA cycle to fix carbon while gamma-Proteobacteria predominantly use the CBB cycle [11]. *Riftia pachyptila* has similar differences in δ^13^C among vent sites, but this is attributed to its endosymbionts shifting between rTCA and CBB cycles [53] rather than changes in the microbial community it consumes. Alvinoconchid gastropods have δ^13^C values that differ by >20‰ among vent fields, which relates to whether epsilon- or gamma-Proteobacteria are the endosymbionts [54]. It is unclear why *Kiwa* sp. epibiont diversity is different between E2 and E9. At other hydrothermal vent locations differences in vent fluid chemical composition influences microbial communities [46], [55] and it may be similar at the ESR vent fields. The difference in carbon fixation appeared to be transferred through *Kiwa* sp. to the predatory stichasterid seastar. Such a predator-prey interaction may also explain the large difference in δ^13^C values between E2 and E9N in *Sericosura* spp. At E2 *Sericosura* spp. were collected from amongst anemones that had δ^13^C values indicative of a mixed carbon source but at E9 they were collected from amongst peltospiroid gastropods dependent on CBB fixed carbon, although *Sericosura* spp. were not observed directly feeding on either anemones or Peltospiroidea sp.

Relatively small differences in stable isotope values were observed among sites in Peltospiroidea sp., *Lepetodrilus* sp. and *Vulcanolepas* sp. To date, Peltospiroidea sp. contains a single strain of gamma-Proteobacteria endosymbiont (K. Zwirglmaier unpublished data), which means spatial differences in δ^13^C and δ^34^S are unlikely to be the result of differences in the type of endosymbiont [55]. The differences were potentially a result of site-specific variations in the δ^13^C_DIC_ and inorganic δ^34^S values used by the endosymbionts during chemoautotrophy or physiological temperature-related effects on isotopic discrimination. Small differences among sites for the grazer *Lepetodrilus* sp. and suspension feeder *Vulcanolepas* sp. are harder to explain because of the various factors that are likely to influence their food source. δ^13^C values indicated these organisms consume a mixed diet of free-living microbes and particulate material. However, differences in δ^13^C values within sites may be related to the organism’s distribution within the vent field [56] and in turn the composition of the microbial community [46], the stable isotope values of the inorganic substrate used during chemoautotrophy [57] and temperature effects on trophic discrimination. *Lepetodrilus* sp. and *Vulcanolepas* sp. were collected from single points within each vent site and, therefore, it is not clear whether the difference in stable isotope values among sites is greater or less than that within sites.

Because of the snap-shot nature of this study, it is difficult to identify factors that caused the spatial differences in the *Kiwa* sp. epibiont communities that resulted in a greater range of δ^13^C values at the E9 sites compared to E2. Higher concentrations of dissolved sulfides in vent fluids may favour the rTCA pathway resulting in increasing numbers of organisms with δ^13^C values greater than −16‰ [12]. On the ESR, E9 has higher hydrogen sulfide and lower chloride concentrations than E2 meaning that there are greater concentrations of available gases for microbial primary production due to phase separation [6], [21]. Higher concentrations of reduced compounds and gases may be one of the drivers of the differences in trophic structure at the ESR vents. However, hydrothermal vent communities also undergo changes in community composition with age [58] and fluctuating hydrothermal activity [59], which will have an effect on trophic structure. As data presented here were obtained concurrently with the discovery of the new biogeographical province it is not possible to determine whether the communities at E2 and E9 represent different successional stages, are a product of varying chemistry or a mix of such processes.

### Conclusion

Trophic structure differed substantially between the E2 and E9 vents fields, and only slightly between E9N and E9S. δ^13^C_DIC_ of the end-member fluid and diffuse flow samples were similar among the sites but large differences in the δ^13^C values of some vent macroconsumers indicated spatial variations in the way microbes were fixing carbon at the base of the food chain. δ^13^C values >−13‰ at the E9N and E9S suggest that the relative contribution to the macroconsumer food web of carbon fixed via the rTCA cycle is likely to be greater than at E2. The greater range of δ^34^S values at E2 and E9S indicated a potentially greater influence of epipelagic photosynthetic primary production than at E9N. The greater contribution of rTCA fixed carbon at the E9 vent field may ultimately be related to differences in vent fluid, but more work is required to link vent fluid chemistry with microbial primary production and the related trophic structure at hydrothermal vents.
